# Small Bowel Obstruction due to Intestinal Xanthomatosis

**DOI:** 10.1155/2015/231830

**Published:** 2015-06-17

**Authors:** L. E. Barrera-Herrera, F. Arias, P. A. Rodríguez-Urrego, M. A. Palau-Lázaro

**Affiliations:** ^1^Pathology and Clinical Laboratory Department, Fundación Santa Fe de Bogotá University Hospital, Calle 119, No. 7–75, Bogotá 110111, Colombia; ^2^Surgery Department, Fundación Santa Fe de Bogotá University Hospital, Calle 119, No. 7–75, Bogotá 110111, Colombia; ^3^School of Medicine, Universidad de los Andes, Carrera 1, No. 18A-12, Bogotá 111711, Colombia

## Abstract

Vast majority of bowel obstruction is due to postoperative adhesions, malignancy, intestinal inflammatory disease, and hernias; however, knowledge of other uncommon causes is critical to establish a prompt treatment and decrease mortality. Xanthomatosis is produced by accumulation of cholesterol-rich foamy macrophages. Intestinal xanthomatosis is an uncommon nonneoplastic lesion that may cause small bowel obstruction and several cases have been reported in the English literature as obstruction in the jejunum. We report a case of small intestinal xanthomatosis occurring in a 51-year-old female who presented with one day of copious vomiting and intermittent abdominal pain. Radiologic images revealed jejunal loop thickening and inflammatory changes suggestive of foreign body obstruction, diagnostic laparoscopy found two strictures at the jejunum, and a pathologic examination confirmed a segmental small bowel xanthomatosis. This case illustrates that obstruction even without predisposing factors such as hyperlipidemia or lymphoproliferative disorders.

## 1. Introduction

Small bowel obstruction corresponds to approximately 20% of patients who present with acute abdomen and are admitted for surgery [1]. Among the different causes of small bowel obstruction (SBO), 60% are due to postoperative adhesions, followed by malignancy, intestinal inflammatory disease (Crohn's disease), and hernias [2]. SBO is difficult to recognize preoperatively and is associated with significant mortality. About 5.5% present with strangulation [3], making surgery the essential treatment to prevent further complications. Xanthomatosis is characterized by accumulation of lipid-laden, foamy macrophages resulting in nodule formation and commonly involving the skin [4]. In the gastrointestinal tract, stomach is the most frequent location; however, cases involving esophagus [5], small bowel [6, 7], ileocecal valve [8], colon [9–12] and rectum [13], tendons, bladder, and prostate have been described [14]. We report a case of SBO due to intestinal xanthomatosis.

## 2. Clinical Summary

**Figure 1:**
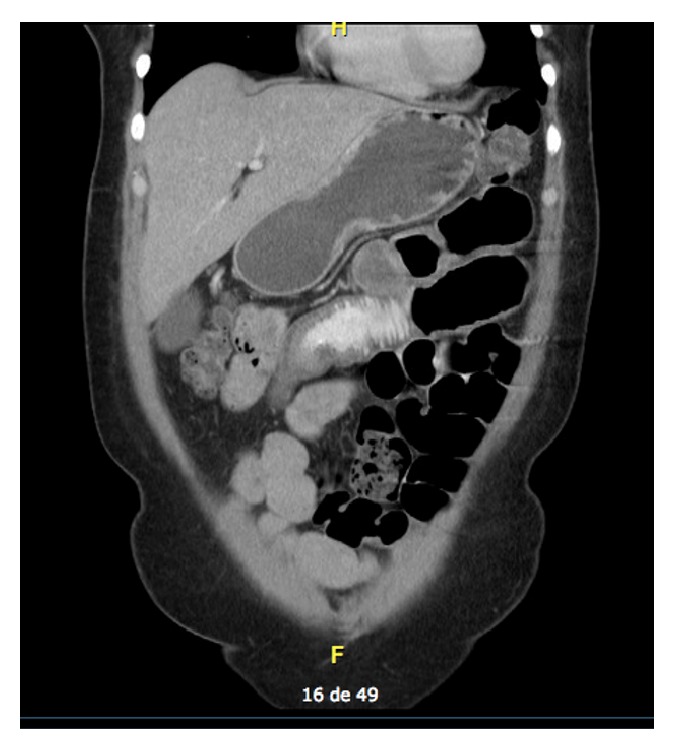
CT imaging presenting adynamic ileus with jejunal loop thickening.

A 51-year-old female presented with one day of copious vomiting and intermittent abdominal pain. Her clinical history was significant for hypertension treated with Losartan and Hydrochlorothiazide. Physical examination revealed mild dehydration and diffuse abdominal pain with no peritoneal irritation signs. CBC showed normal leucocyte count 4.58 × 10^3^/*μ*L (5–10 × 10^3^/*μ*L) with neutrophilia 84% (30–40%); CT scan of the abdomen (Figure 1) revealed an adynamic ileus with jejunal loop thickening and inflammatory changes suggestive of foreign body obstruction. The patient was taken to diagnostic laparoscopy which revealed two strictures at the jejunum. Small bowel resection with anastomosis was performed and the adherences were liberated. The patient had an uneventful recovery.

## 3. Pathologic Findings

**Figure 2:**
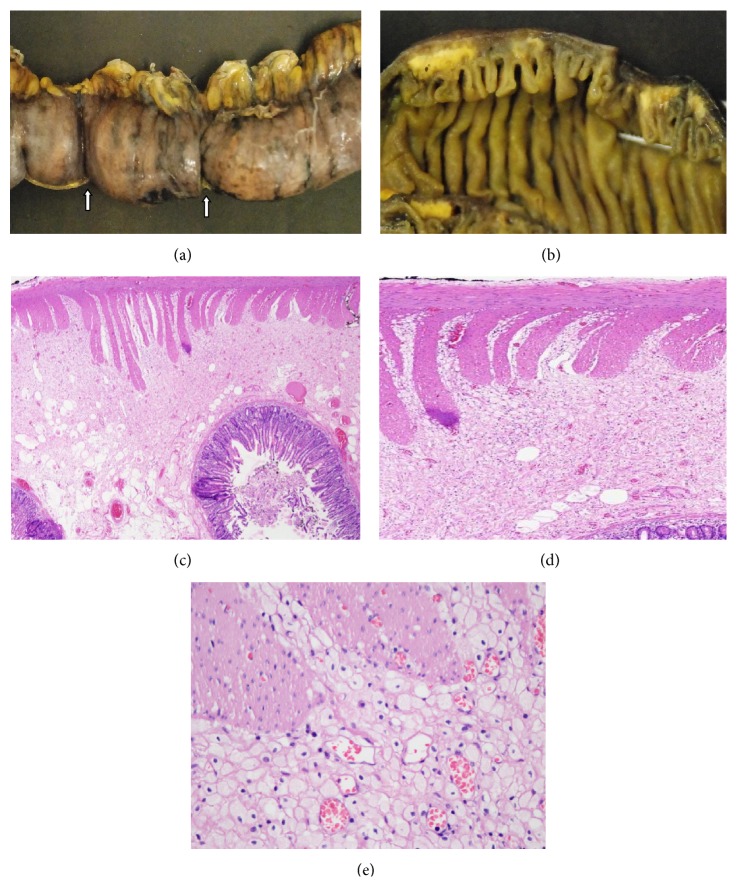
(a) Small bowel segment with two areas of stricture (see arrows). (b) Full thickness small bowel wall with submucosal yellowish, (c) H&E 40x, (d) 100x, and (e) 400x xanthomatous histiocytes involving the submucosa and muscularis propria.

Gross examination revealed a 16 cm segment of jejunum with circumference of 5 cm. The serosal surface had two strictures (Figure 2(a)). On opening, two areas of narrowed lumen with a circumference of 2 cm were noted. The wall at the site of strictures had a yellow mural discoloration that involved the submucosa (Figure 2(b)).

**Figure 3:**
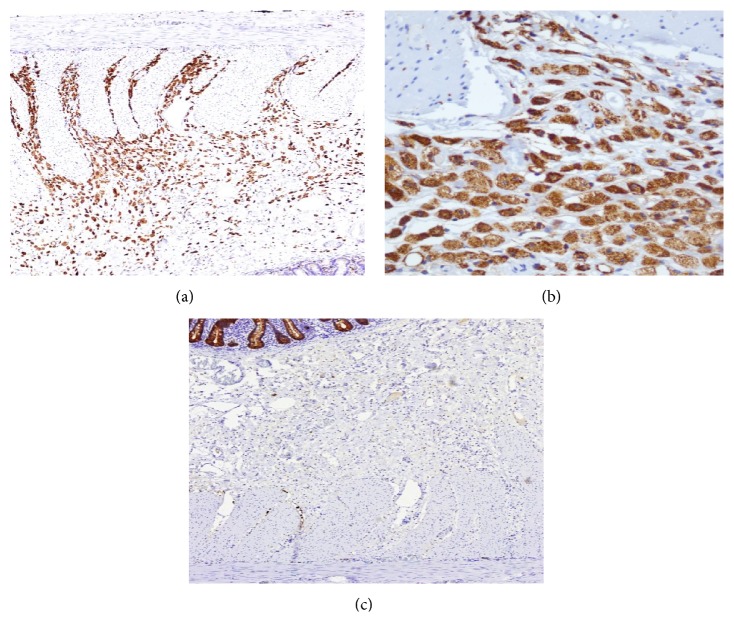
(a) (100x), (b) (400x) histiocytes positive for CD68 and (c) (100x) negative cytokeratin AE1/AE3.

Microscopic examination revealed an interstitial diffuse infiltrate of foamy histiocytes that involved mainly the submucosa and infiltrate focally the muscularis propria pushing the mucosa upwards (Figures 2(c) and 2(d)). Sections from mesenteric vessels showed moderate atherosclerosis with medial calcification. Special stains for PAS and PAS-D, Gomori, methenamine silver, Ziehl Neelsen, and Gram were negative for microorganisms. Immunohistochemistry studies were positive for CD68 and negative for cytokeratin AE1/AE3 (Figure 3) confirming a histiocytic origin. Final diagnosis of segmental small bowel xanthomatosis was made.

## 4. Discussion

Xanthomas are defined as local accumulation of cholesterol-rich foamy macrophages in tissue; intestinal xanthomatosis is considered an unusual nonneoplastic lesion that may cause obstruction and can present as a mass-like lesion mimicking malignant tumor obstruction due to prominent fibrosis and inflammation [15], as in our case. The etiologies of intestinal xanthomatosis not associated with predisposing conditions such as hyperlipidemia or lymphoproliferative lesions have not been settled and one of the theories related to its development states that an insult generates destruction of cells in the mucosa or submucosa with subsequent ingestion of lipid-containing debris by histiocytes which then persist as foamy cells [16]. Gastrointestinal xanthomas are rare and the stomach is the most frequent described location [4].

Although xanthomas are commonly seen in patients with dyslipidemias or other conditions such as previous chemotherapy [16], radiotherapy [6], and infection (disseminated mycobacterium avium-intracellular and cytomegalovirus colitis) in immunosuppressed patients (AIDS) [8], our case did not have history of any of these treatments, immunosuppression, and known history of hypercholesterolemia (lipid profile not available) and no skin xanthomas were found on physical examination. Review of the literature about intestinal xanthomatosis shows that this entity is predominantly found incidentally during endoscopy; however, among the symptomatic cases, some presented with obstruction and strictures/stenosis that caused dysmotility of the intestinal musculature [6]. Also some cases had presented as small bowel pseudotumors [16]; the reported symptomatic patients by Delacruz et al. had either hyperlipidemia or lymphoproliferative disorders [4]. Neither of these conditions was found in this case.

Gastrointestinal xanthomas are composed of cells with abundant foamy cytoplasm containing lipid characteristically positive for CD68 with no mucin or pigment deposition. Microscopically, the differential diagnosis includes poorly differentiated carcinoma, storage diseases, infections (Whipple disease, mycobacterium, and AIDS), macroglobulinemia, and muciphages [4]. In our case, the negativity for cytokeratin AE1/AE3 ruled out carcinoma; the negativity for special stains such as Gram, Ziehl Neelsen, Gomori methenamine silver, and PAS and PAS-D ruled out infections and mucin deposition. Besides clinically the patient did not have symptoms for storage diseases and/or macroglobulinemia.

## 5. Conclusion

Intestinal xanthomatosis is a rare entity but it should be considered as one of the etiologies of clinically significant obstruction and must be included even if the patient has no history of hyperlipidemia or lymphoproliferative disorder. In order to implement the best treatment options if diagnosed, clinicians must extend workup to rule out lipid storage diseases, dyslipidemia, lymphoproliferative disorders, and infections [17].
